# Renal Failure Affects the Enzymatic Activities of the Three First Steps in Hepatic Heme Biosynthesis in the Acute Intermittent Porphyria Mouse

**DOI:** 10.1371/journal.pone.0032978

**Published:** 2012-03-06

**Authors:** Carmen Unzu, Ana Sampedro, Eliane Sardh, Itsaso Mauleón, Rafael Enríquez de Salamanca, Jesús Prieto, Eduardo Salido, Pauline Harper, Antonio Fontanellas

**Affiliations:** 1 Gene Therapy and Hepatology Area, Centre for Applied Medical Research (CIMA), University of Navarra, Pamplona, Spain; 2 Department of Internal Medicine, Karolinska Institutet, Stockholm Söder Hospital, Stockholm, Sweden; 3 Porphyria Centre Sweden, Department of Laboratory Medicine, Karolinska Institutet, Karolinska University Hospital, Stockholm, Sweden; 4 Research Center, Hospital Universitario 12 de Octubre, Universidad Complutense, Madrid, Spain; 5 Centre for Biomedical Research on Rare Diseases (CIBERER), University Hospital of Canarias, University of La Laguna, La Laguna, Spain; University of Cordoba, Spain

## Abstract

Chronic kidney disease is a long-term complication in acute intermittent porphyria (AIP). The pathophysiological significance of hepatic overproduction of the porphyrin precursors aminolevulinate acid (ALA) and porphobilinogen (PBG) in chronic kidney disease is unclear. We have investigated the effect of repetitive acute attacks on renal function and the effect of total or five-sixth nephrectomy causing renal insufficiency on hepatic heme synthesis in the porphobilinogen deaminase (PBGD)-deficient (AIP) mouse. Phenobarbital challenge in the AIP-mice increased urinary porphyrin precursor excretion. Successive attacks throughout 14 weeks led to minor renal lesions with no impact on renal function. In the liver of wild type and AIP mice, 5/6 nephrectomy enhanced transcription of the first and rate-limiting ALA synthase. As a consequence, urinary PBG excretion increased in AIP mice. The PBG/ALA ratio increased from 1 in sham operated AIP animals to over 5 (males) and over 13 (females) in the 5/6 nephrectomized mice. Total nephrectomy caused a rapid decrease in PBGD activity without changes in enzyme protein level in the AIP mice but not in the wild type animals. In conclusion, high concentration of porphyrin precursors had little impact on renal function. However, progressive renal insufficiency aggravates porphyria attacks and increases the PBG/ALA ratio, which should be considered a warning sign for potentially life-threatening impairment in AIP patients with signs of renal failure.

## Introduction

Acute intermittent porphyria (AIP, OMIM 176000) is an inherited metabolic disease characterized by partial deficiency of hepatic porphobilinogen deaminase (PBGD). The disease is inherited in an autosomal dominant manner and is the most common of the acute porphyrias [1]. The dominant clinical feature is characterized by acute attacks of abdominal pain, hypertension and neurovisceral and circulatory disturbances, a condition which if untreated may become life-threatening. An inherited deficiency of PBGD is not sufficient for the symptoms to appear. Acute attacks can be induced by various drugs, nutritional factors and hormonal changes [2]. Drugs metabolized by CYP450, such as phenobarbital, greatly increase hepatic heme demand and result in the up-regulation of hepatic aminolevulinate synthase (ALAS1), increasing the production of porphyrin precursors and precipitating the attack. Advances in medical care and self-care have improved the prognosis for symptomatic patients [3]. Still, some patients develop recurrent crises or progressive disease with disabling neurological dysfunction and/or renal failure.

Chronic kidney disease may occur as a long-term complication of symptomatic disease in acute porphyrias, leading to vascular complications, progression of peripheral neuropathy and eventually need for dialysis. Chronic arterial hypertension and renal impairment become more common after middle age in AIP, especially in patients with frequent porphyric attacks [4], [5]. Limited information is available on the characteristics and pathogenesis of renal disease in this patient group and little is known about the association between renal damage and acute porphyrias. The disease may be accompanied by electrolyte abnormalities. Hyponatremia is common during the acute attack and may be due to inappropriate secretion of antidiuretic hormones. The increased urinary excretion of catecholamines during an acute attack suggests up regulation of the sympathetic nervous system and may contribute to the etiology of renal damage. Chronic hypertension in AIP has an estimated incidence of 36–55% [3]. However, long-term treatment with modern antihypertensive drugs has minor the repercussion on renal function.

There are few studies in which biopsies have been performed in patients with AIP. In one large population study [4], 16 patients were found with renal impairment with no other cause than AIP. Nine of the 10 patients that underwent renal biopsy had hypertension. The biopsies showed varying degrees of nephrosclerosis, moderate tubular atrophy and interstitial fibrosis and vessel wall thickening. Other few authors reported nephrosclerosis and shrunken kidney more in accordance to hypertension damage [6], [7], [8]. Finally, renal biopsy data from patients without hypertension or glomerular lesions but with features of tubulointerstitial disease suggest an enhanced susceptibility to the nephrotoxic effects of porphyrin precursors and porphyrins [9], [10]. Both types of damages directly or indirectly may point to active AIP, with or without frequent hemin administration [11], as an important factor causing renal disease. Of interest, a selective accumulation and excretion of PBG has been found in most porphyric patients with renal failure [9], [11] and an increased PBG/ALA ratio was proposed as a warning sign associated to changes in the renal filtration.

In the present work, using an AIP mouse model [12], we studied the effects on renal function of repetitive acute attacks, as well as the effects of partial or total nephrectomy causing renal insufficiency. This report is the first *in vivo* study to address the liver-kidney interaction during acute porphyric attacks. Although the mouse is a predictive model for AIP, extrapolation to human disease is considered and discussed in the paper.

## Results

### Successive induced biochemical attacks increased porphyrin precursor accumulation with no impact on renal function

**Figure 1:**
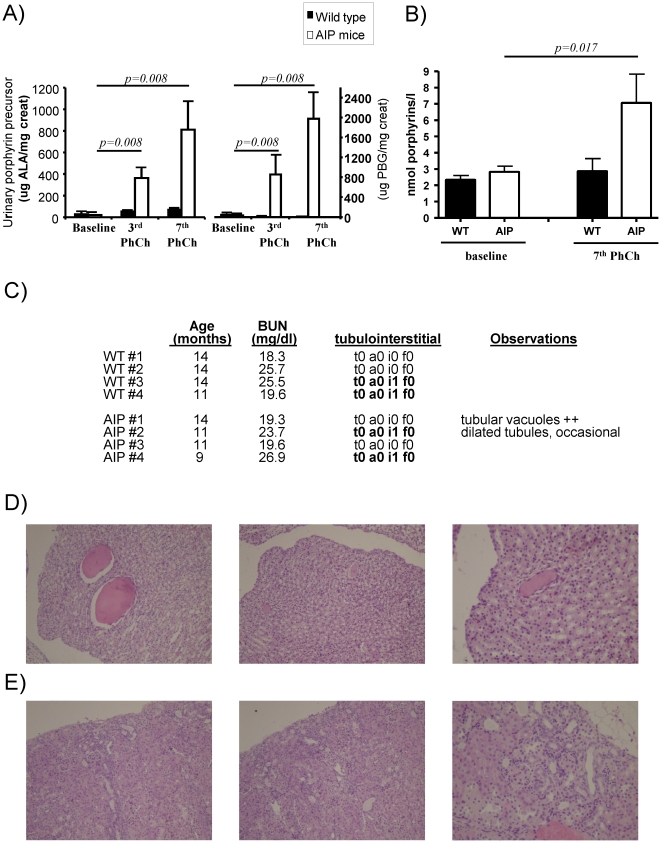
Lack of glomerular, tubulointerstitial or vascular damage in acute intermittent porphyria mice after sustained urinary porphyrin precursors and porphyrin excretion induced by phenobarbital challenge. The effect of repetitive acute attacks on renal function was assayed in four AIP male mice aged from 9 to 14 months. Seven consecutive phenobarbital challenges were administered at intervals of two weeks in order to maintain high hepatic production and high flow of both porphyrin precursors and porphyrin throughout the kidney. Four age-matched wild type male animals were used as controls. After the fourth consecutive dose of each phenobarbital challenge AIP mice developed a biochemical attack, characterized by A) high levels of urinary porphyrin precursors and B) porphyrin accumulation. C) Blood urea nitrogen (BUN) and individual pathological analysis of kidney necropsies. Histological analysis was performed in renal samples taken three days after the last phenobarbital challenge. Prognostic scores in histological analysis of renal biopsies were analysed by a trained pathologist (ES) as described in Methods. D) Light micrographs of kidney sections showed relatively innocuous tubular dilatation in one AIP animal. Another AIP animal showed diffuse cortical atrophy E), which was more prominent in the subcapsular region. Magnification ×200. The Wilcoxon matched pairs test was used for comparison. The null hypothesis was rejected when p≤0.05. WT, wild type; AIP, Acute Intermittent Porphyria.

The AIP mouse model exhibits a 70% loss of PBGD activity in the liver and replicates the drug-precipitated biochemical abnormalities of acute porphyria in humans [12]. The administration of four consecutive intraperitoneal daily doses of phenobarbital massively increased urinary excretion of heme precursors and decreased motor function. Histopathological findings include axonal neuropathy and decrease nerve conduction with aging [12] [13]. In order to study the nephrotoxic effects of porphyrin precursors and porphyrins, seven successive attacks over 14 weeks were induced in one year old male AIP mice. Matched wild type mice were used as controls. As expected, phenobarbital challenge increased urine levels of ALA, PBG (Figure 1A) and porphyrins (Figure 1B) in AIP mice. The mean excretion of PBG (µg per mg creatinine) was twice as high as the excretion of ALA (Figure 1A), i.e. PBG/ALA ratio 2. The daily amount of PBG and ALA excreted in the urine after the 7^th^ phenobarbital challenge was significantly higher than that after the 3^rd^ challenge. Urinary porphyrin precursors and porphyrins were unchanged within the normal range in wild type animals (Figure 1A, 1B). In AIP mice, successive acute attacks did not modify renal function as measured by blood urea nitrogen values (Figure 1C).

These animals were sacrificed three days after the last phenobarbital dose of a challenge. The ALAS1 mRNA level was significantly increased in AIP mice when compared with wild type mice, 2.3±1.2 *vs* 1.0±0.9 Arbitrary Units, respectively; *p* = 0,0498, one-tailed unpaired *t*-test with Welch's correction.

Histological analysis of kidney tissues from AIP mice and age-matched wild type animals showed lack of porphyrin deposits and no vascular or tubulointerstitial damage (Figure 1C). Half of the animals from each group exhibited focal accumulations of mononuclear inflammatory cells, mostly in the perivascular space. Light micrographs of kidney sections showed relatively innocuous tubular dilatation in one AIP animal (Figure 1D) and diffuse cortical atrophy in the subcapsular region in another AIP animal (Figure 1E). These changes had no impact on renal function and can occur as a senile change in old wild type animals. These results show that high excretion of porphyrin precursors and porphyrins have little impact on renal function. Mild degrees of renal lesions can occur as a senile change in old animals and seem unrelated to acute attacks of porphyria.

### Partial nephrectomy raised urinary PBG/ALA ratio in porphyric animals

**Figure 2:**
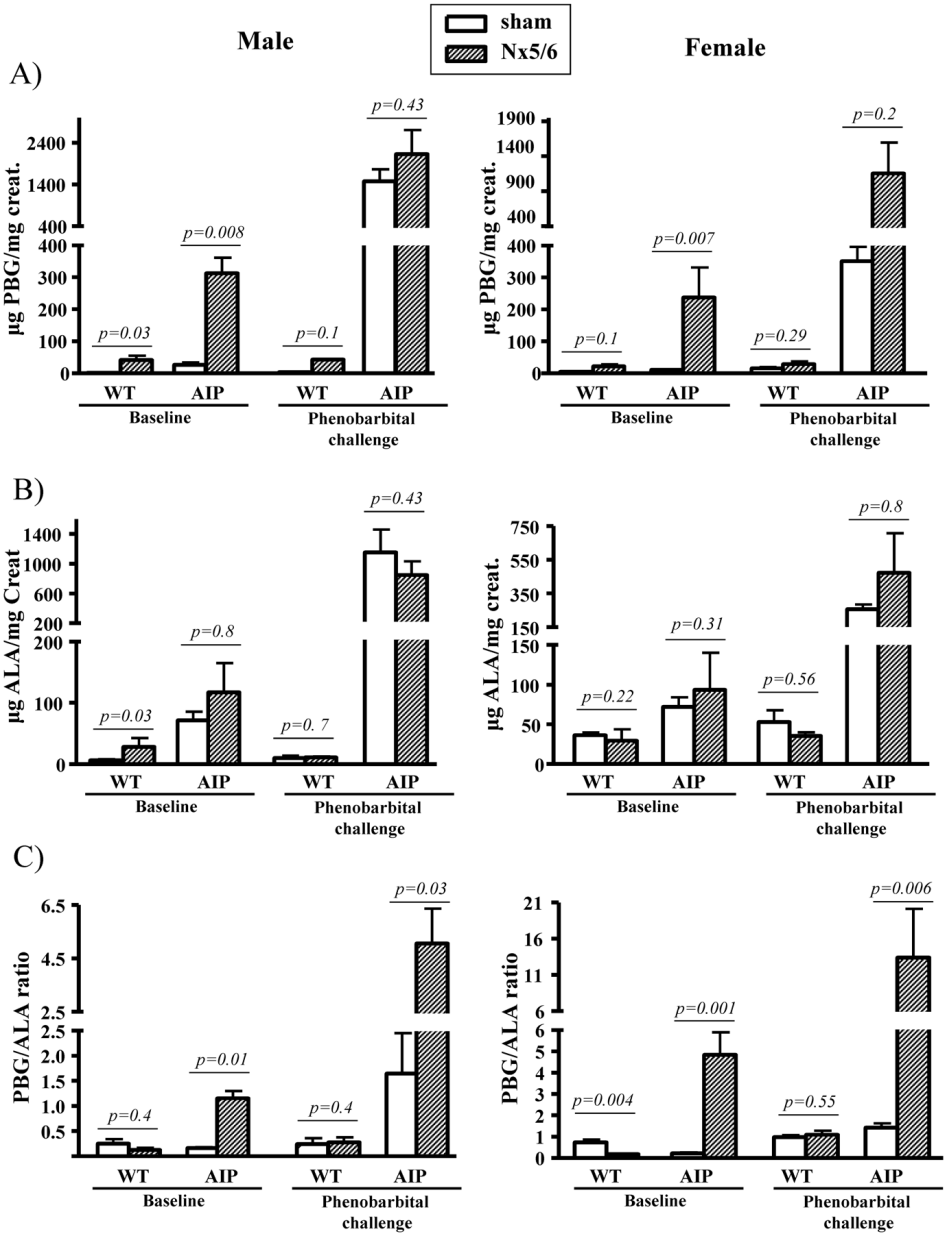
Porphyrin precursor excretion in wild type and AIP mice suffering from different degrees of renal insufficiency. The effect of five-sixth nephrectomy on urine excretion of aminolevulinate acid (ALA) and porphobilinogen (PBG) was compared with respect to urinary excretion in sham-operated mice. Urine levels of ALA and PBG were also measured in these animals after phenobarbital challenge that induces a biochemical attack of porphyria specifically in AIP mice. A) Urinary PBG excretion. B) Urinary ALA excretion. C) Urinary PBG/ALA ratio. The non-parametric Mann–Whitney U-test was used for comparison of two groups. Nx5/6, 2/3 nephrectomy of the left kidney and extirpation of the right kidney. The null hypothesis was rejected when p≤0.05. WT, wild type; AIP, Acute Intermittent Porphyria.

In a second study, five-sixth nephrectomy was performed in adult wild type and AIP mice after 2/3 nephrectomy of one kidney and extirpation of the other. Other cohorts of mice were sham-operated. Heme precursor excretion were measured before and after phenobarbital challenge (Figure 2A–C and Table 1). Renal insufficiency caused by 5/6 nephrectomy *per se* increased in the AIP mice the urinary PBG excretion (Figure 2A, baseline) in male and female, *p* = 0.008 and *p* = 0.007 respectively, compared to values in sham operated AIP mice. No changes were observed in urinary ALA (Figure 2B, baseline) and porphyrin excretion (Table 1). As expected, phenobarbital challenge exacerbated ALAS1 up-regulation and high levels of both porphyrin precursors and porphyrins were found in the urine (Figure 2A and 2B and Table 1), both in sham operated and 5/6 nephrectomized AIP mice of both sexes.

**Table 1:** Serum cystatine and porphyrin levels in wild type and AIP mice with different degrees of chronic renal failure.

Parameter	Group	Phenobarbital administration	WT male				AIP male				WT female				AIP female			
**Serum cystatine (ng/ml)**	Sham	-	337	±	73		297	±	134		291	±	129		276	±	128	
	Nx 2/6	-	445	±	112		560	±	58	*	424	±	132	*	537	±	26	*
	Nx 5/6	-	2164	±	791	*	1157	±	982	*	2075	±	1050	*	1870	±	1075	*
	Nx 6/6	-	3494	±	570	*	3864	±	687	*	3004	±	656	*	3683	±	731	*
**Serum porphyrines (nmol/l)**	Sham	No	3.0	±	0.7		3.6	±	0.7		2.7	±	0.5		3.4	±	0.5	
	Sham	Yes	3.2	±	0.5		6.6	±	2.6		3.3	±	1.1		7.6	±	2.4	*
	Nx 5/6	Yes	7.4	±	2.4		9.5	±	2.8	*	5.9	±	3.7		16.8	±	18.7	*
	Nx 6/6	Yes	10.9	±	6.2		62	±	41	*	8.7	±	3.8		20.4	±	8.5	**
**Hepatic porphyrines (nmol/g prot.)**	Sham	No	0.6	±	0.1		2.5	±	0.5		0.7	±	0.8		2.2	±	0.5	
	Sham	Yes	1.0	±	0.1		2.9	±	0.3		0.8	±	0.3		2.2	±	0.04	
	Nx 5/6	Yes	0.5	±	0.2		1.6	±	1.0		0.7	±	0.3		2.8	±	1.35	
	Nx 6/6	Yes	0.9	±	0.2		2.9	±	0.4		1.0	±	0.4		2.4	±	0.3	
**Urine porphyrines (nmol/l)**	Sham	No	23	±	7		39	±	8		15	±	4		38	±	16	
	Sham	Yes	16	±	9		344	±	141	**	33	±	18		154	±	90	*
	Nx 5/6	No	20	±	3		37	±	27		40	±	6		48	±	8	
	Nx 6/6	Yes	8	±	2		196	±	95	**	39	±	56		203	±	147	*

WT, wild type; AIP, Acute Intermittent Porphyria, Nx5/6, 2/3 nephrectomy of the left kidney and extirpation of the right kidney; Nx 6/6, total nephrectomy. Mann–Whitney U-test was used for comparison of two groups (* P<0.05, ** P<0.01 vs Sham-operated group with no phenobarbital administration). The null hypothesis was rejected when P≤0.05.

Phenobarbital challenge underlined the selective accumulation of PBG in partially nephrectomized animals, as measured by the increased PBG/ALA ratio (Figure 2C). The urinary PBG/ALA ratio in wild type and AIP mice with conserved renal function was under 1.0. In the 5/6 nephrectomized AIP mice, the PBG/ALA ratio increased above 1.0, and phenobarbital challenges further increased this ratio from 1 to 5 in males (Figure 2C, left) and from 5 to 13 in AIP females (Figure 2C, right). Of note, five-sixth nephrectomy did not result in porphyrin accumulation in the serum (Table 1), suggesting that porphyrins were readily filtered by the remnant kidney. The increased concentration of urinary porphyrin observed in sham operated and nephrectomized AIP mice after phenobarbital challenge, is a consequence of nonenzymatic condensation of PBG to uroporphyrin I [14].

**Figure 3:**
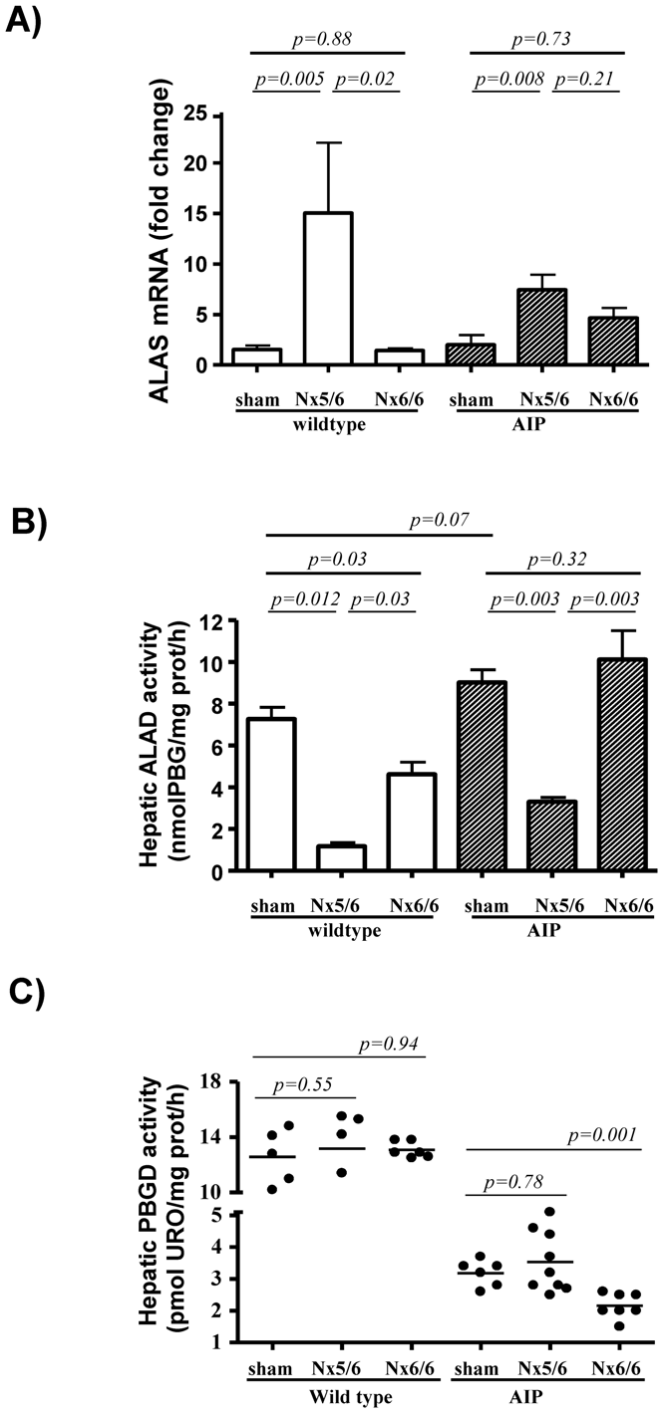
Expression profile of hepatic ALAS, ALAD and PBGD in female wild type and AIP mice suffering from different degrees of renal insufficiency. A group of 4 wild type and 9 AIP female mice were subjected to 5/6 nephrectomy (5/6 Nx) while complete bilateral nephrectomy was performed in 6 wild type and 7 AIP mice. A group of 5 wild type and 6 AIP female animals were sham-operated. A) Quantitative real-time PCR analysis of ALAS mRNA from liver samples. The amount of each transcript was expressed according to the formula 2^ΔCt(Actin)−ΔCt(gene)^, where ΔCt is the point at which the fluorescence rises appreciably above background fluorescence. B) ALAD and C) PBGD activities measured in the liver of mice taken at sacrifice. The non-parametric Mann–Whitney U-test was used for comparison of two groups. Nx5/6, 2/3 nephrectomy of the left kidney and extirpation of the right kidney; Nx 6/6, total nephrectomy. ALAS, hepatic aminolevulinate acid synthase; ALAD, aminolevulinate acid dehydratase; AIP, Acute Intermittent Porphyria.

**Figure 4:**
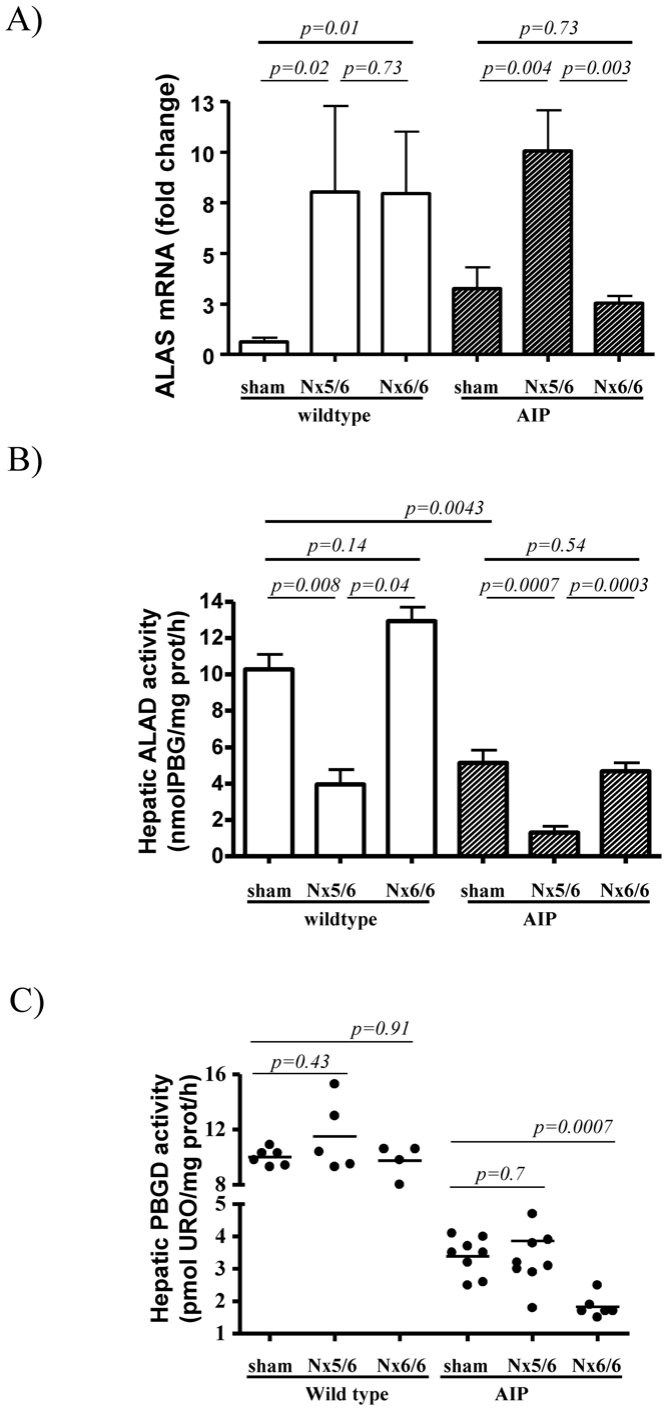
Expression profile of hepatic ALAS, ALAD and PBGD in male wild type and AIP mice suffering from different degrees of renal insufficiency. A group of 5 wild type and 8 AIP male animals were subjected to 5/6 nephrectomy (5/6 Nx) while complete bilateral nephrectomy was performed in 4 wild type and 6 AIP mice. A group of 6 wild type and 8 AIP male animals were sham-operated. A) Quantitative real-time PCR analysis of ALAS mRNA, B) enzyme ALAD and C) PBGD activities measured in the liver of wild type and AIP male animals. The non-parametric Mann–Whitney U-test was used for comparison of two groups. Nx5/6, 2/3 nephrectomy of the left kidney and extirpation of the right kidney; Nx 6/6, total nephrectomy. ALAS, hepatic aminolevulinate acid synthase; ALAD, aminolevulinate acid dehydratase; AIP, Acute Intermittent Porphyria.

The genetic expression and activity of the three first enzymatic steps of the heme synthesis pathway was assessed in the liver of these nephrectomized mice 24 h after the last phenobarbital dose are shown in Figure 3 (female) and Figure 4 (male). The 5/6 nephrectomy increased ALAS1 mRNA levels (Figure 3A and 4A) and reduced ALA dehydratase (ALAD) activity in the liver (Figure 3B and 4B) in both wild type and AIP mice. As there were significant sex differences in hepatic ALAS1 mRNA levels and ALAD activity the results are presented separately.

No changes were found in the activity of the PBGD enzyme in wild type animals (Figure 3C and 4C). The genetically deficient PBGD activity was not further decreased by 5/6 nephrectomy (Figure 3C and 4C). The mRNA levels for ALAD and PBGD found in the liver substantiated the respective hepatic enzyme activity obtained in these animals (Figure S1). These data suggested that ALAS1 over-expression induced by partial nephrectomy, increases the synthesis of heme precursors, causing a selective accumulation of PBG, the substrate of the deficient enzyme, PBGD. ALA was not increased and suggested that ALAD can not became a rate-limiting step even after a marked decrease subsequent to partial nephrectomy.

In male animals, sham-operated AIP mice showed 3-fold increased ALAS1 mRNA levels when compared with wild type animals (Figure 4A). These data (obtained from animals sacrificed three days after last phenobarbital dose) suggested that transcriptional activation of the hepatic ALAS1 gene in response to xenobiotic challenge in the AIP mice remained active over a longer time in males compared to females. Differences in the duration of ALAS1 activation during phenobarbital challenge correlates with the higher excretion of porphyrin precursors in males compared to females (Figure 2). However, 5/6 nephrectomy exacerbated a selective accumulation of PBG in both females (from 5 before to 13 after 5/6Nx) and males (from 1 before to 5 after 5/6Nx) (Figure 2C).

The remnant kidney pole from AIP mice illustrated normal glomerular architecture without vascular atrophy or heme precursor deposits (Figure S2). Histology also showed lack of tubulointerstitial damage and inflammatory cell infiltrate. These data suggested that the presumably noxious effect of porphyrin precursors and porphyrins on renal parenchyma were not intensified after reduction of renal function in the AIP mice model during the observation time.

### Total nephrectomy induced a rapid decrease in PBGD activity in AIP mice

In order to measure the impact of terminal renal disease on the hepatic heme synthesis pathway, a total nephrectomy was performed in another cohort of mice during a phenobarbital challenge. Lack of renal function produced a rapid accumulation of heme precursors in serum of the AIP mice (Table 1). Ten hours after nephrectomy, hepatic transcription levels of ALAS1 were found to be unchanged in both female (Figure 3A) and male AIP mice (Figure 4A) when compared with sex-matched wild type animals. Of interest, a rapid decrease in hepatic PBGD activity was observed exclusively in AIP mice (Figure 3C and 4C), with no changes in transcriptional activity (Figure S1) or enzyme protein level (Figure S3). It may be assumed that the observed aggravation of PBGD deficiency may be a consequence of further PBGD-inhibition caused by the large substrate accumulation caused by impaired glomerular filtration.

## Discussion

Limited information is available on the association between acute intermittent porphyria and kidney failure. The development of end stage renal disease is a devastating complication in AIP patients with chronic active disease, leading to unavoidable vascular complications, dialysis treatment, progression of peripheral motor neuropathy and, occasionally, respiratory failure [15]. Such patients may also suffer from cutaneous bullous lesions resembling PCT [11], [16]. Current treatment of acute attacks involving intravenous administration of hemin and a high-carbohydrate diet only has a transitory effect and cannot prevent accumulation of porphyrin precursors and porphyrins in-between cures [11]. There are no reports of longstanding follow-up of renal function in patients afflicted by recurrent acute attacks and only few studies have reported results of kidney biopsies in AIP patients with chronic disease [4], [9], [10].

The leading hypothesis is that the porphyric state may progressively damage the kidneys sufficiently to cause renal failure. It has been suggested that the presence of excessive amounts of porphyrin precursors and porphyrins causes cytotoxic or vasospastic renal vascular lesions leading to glomerulonephritis and tubulointerstitial nephritis [9], [10], [11]. Repeated hemin therapy may also contribute to renal damage [17], [18]. The high accumulation of porphyrin precursors, mainly ALA, has been proposed as a cause of renal toxicity by oxidative stress and lipid peroxidation in microsomal and mitochondrial membranes [10], [19]. ALA accumulation is thought to be related to increased oxidative stress [20]. However, increased incidence of primary liver cancer in Swedish and French patients suffering from AIP [21], [22], [23] has not directly pointed to a correlation between disease activity and increased levels of porphyrin precursors, i.e. oxidative stress. Moreover, there is no evidence of a high incidence of renal cancer in AIP patients, at least not in the Swedish and Danish cohorts [21]

In our *in vivo* model, acute attacks were periodically induced by phenobarbital challenge causing intermittent accumulation of porphyrin precursors and porphyrins over a period of 3 months failed to show any important impact on renal function and histology. Probably the toxic effects of porphyrin precursors and porphyrins on the kidney need extended periods of time to significantly alter renal physiology, as occurs in a small number of patients who develop chronic AIP disease characterized by a relatively constant high excretion of porphyrin precursors throughout years [11], [24]. However, most of the AIP-patients with frequent acute attacks do not develop end stage renal disease, which suggests that other key factors are involved [25].

Partial nephrectomy induced the hepatic ALAS1 in both wild type and AIP mice. The up-regulated heme biosynthesis in the AIP mice with genetically deficient PBGD activity gave rise to a selective accumulation of PBG after phenobarbital challenges, leading to an increase in the urinary PBG/ALA ratio. The increased PBG/ALA ratio could not be related to decreases in glomerular filtration since the excretion of molecules such as porphyrins, were not impaired by 5/6 nephrectomy. Thus our data demonstrate that renal insufficiency exacerbated the acute porphyric state shown biochemically by the selective accumulation of PBG, the substrate of the deficient enzyme PBGD. Thus, under conditions of ALAS1 up-regulation, as after 5/6 nephrectomy, the already deficient PBGD enzyme in the liver of the AIP mice may become further overloaded. In the few cases reported by Miyagi et al. [26], PBGD activity measured in the liver of seriously afflicted AIP patients was found to be very low or undetectable. i.e. not the expected 50% of normal activity described for human AIP. The decreased activity was related to a marked increase in serum PBG, suggesting that the PBG might cause further inhibition of hepatic PBGD.

This hypothesis may be supported by this *in vivo* study using the AIP mouse model. Heme biosynthesis induced by phenobarbital administration was followed by total nephrectomy i.e. abolishment of glomerular filtration of heme precursors. Ten hours after total nephrectomy there was an important inhibition of the already decreased hepatic PBGD activity (but not the protein enzyme). These studies demonstrate that end stage renal insufficiency may aggravate the acute porphyria state, hypothetically by substrate inhibition. Physiologically, in a post-translational step, a dipyrromethane cofactor is assembled to apo-PBGD to become the holoenzyme PBGD [27]. An excess of PBG may alter this process and prevent the formation of active PBGD holoenzyme causing a further drop in PBGD activity [28].

The reduced hepatic ALAD activity associated with renal impairment did not modify ALA accumulation as suggested by unchanged urinary ALA excretions in the AIP mice. In fact, the inhibition of ALAD is only significant for ALA accumulation when important ALAD deficiency is observed. This is the case of the homozygous patients with ALAD deficiency, lead poisoning and hereditary tyrosinemia deficiency where the hepatic ALAD activity is less than 1% of the reported normal activity [29], [30].

In conclusion, consecutive phenobarbital challenge in mice caused slight degrees of renal lesions unrelated to porphyria and indicated that massive porphyrin or porphyrin precursor excretion, or their passage through the kidney, did not modify renal function in AIP mice. However, conclusions obtained in the AIP mouse model cannot be extrapolated to chronic AIP disease and we cannot disregard the potential deleterious effect of high porphyrin and porphyrin precursor accumulation maintained during years. Indeed, once end stage renal disease was established we cannot rule out the deleterious effect of porphyrin accumulation since dialysis membranes display a limit efficiency to filter these molecules [11].

Our results indicate that progressive renal insufficiency in AIP mice may aggravate the acute porphyria state. These data may illustrate the pathophysiology in AIP patients afflicted by recurrent acute attacks and renal failure. This is an extremely vulnerable clinical condition in which the development of severe neuropathic complications is very likely. Even though dialysis membranes are able to clear porphyrin precursors [6], [11], [31], [32], ALA and PBG accumulate during the inter-dialysis period and may be responsible for the progression of nerve damage. Increased PBG is nonenzymatically polymerized to uroporphyrin I, a molecule that is not cleared by dialysis leading to accumulation of serum porphyrins and consequently to photosensitivity skin damage [11].

The increase in PBG/ALA ratio should be considered a warning sign for potentially life-threatening aggravation of the porphyric condition. These patients should be candidates for combined kidney and liver transplantation, in order to correct the primary enzyme deficiency as well as restore renal function. These data and other recent cases have clarified previous concerns and could help to formulate the indications for and the timing of transplantation in AIP.

## Materials and Methods

Animal studies. Experimental protocols were performed according to European Council Guidelines. Acceptable standards of humane animal care and treatment employed in these mice (ref. no. CEEA022-06) and the experimental design of this study (ref. no. CEEA029-09) were approved by the Ethics Committee for Animal Testing of the University of Navarra. AIP mice [12] are compound heterozygotes of two different disruptions of the PBGD gene: T1 (C57BL/6-pbgd^tm1(neo)Uam^) and T2 strain (C57BL/6-pbgd^tm2(neo)Uam^) mutations. To biochemically imitate a human porphyria attack, AIP mice were injected intraperitoneally with increasing doses of phenobarbital (75, 80, 85, 90 mg/kg body weight) for four consecutive days (i.e. phenobarbital challenge). Urines (24-hour) were collected in metabolic cages.

Partial or total nephrectomies were performed in 4- to 6-month old mice of both sexes. Acute renal failure was induced surgically. Mice were anaesthetized and kidneys were exposed by dorsal flank incision. In the 5/6 nephrectomy model, the renal artery was briefly clamped and two thirds of the left kidney (upper and lower poles) were excised, leaving the upper pole renal capsule and adrenal gland intact. One week later, the right kidney was removed after ligation of the renal artery, vein and ureter. A phenobarbital challenge was administered one month after extirpation of the right kidney. One day after the last phenobarbital injection, 24-hour urine and serum samples were collected, animals were sacrificed, and the three first enzymatic steps in hepatic heme biosynthesis were investigated.

In the bilateral nephrectomy groups, both kidneys were removed in the same operation, after ligation of the renal artery, vein and ureters. The nephrectomy was performed after the last dose of phenobarbital challenge and animals were killed 10 hours later. Serum and liver samples were collected at each time.

Biochemical analysis. Renal impairment was estimated by serum blood urea nitrogen levels (Ref. 11489364 216, Roche, Germany) or serum cystatin C concentration (Mouse Cystatin C ELISA, BioVendor GmbH) [33] in nephrectomized animal. Total porphyrins were extracted from serum and liver samples with 1 mol/L HCLO_4_/CH_3_OH (1∶1, vol/vol) and measured in a Spectrofluorometer (LS50B, PerkinElmer, Madrid, Spain). Urinary porphyrin concentration was measured according to Westerlund et al. [34]. Uroporphyrin I solutions (10 nmol/L) were used as a standard. Porphyrin precursors, PBG and ALA, were quantified in 24 h urine samples using a quantitative ion exchange column method (BioSystems SA, Barcelona, Spain) and measured by spectrophotometry (Ultrospc 3000, Pharmacia Biotech, Buckinghamshire, UK) at 555 nm. The hepatic activity of aminolevulinate dehydratase was determined by spectrophotometry [35]. PBGD activity was measured as described [13].

RNA extraction and hepatic liver enzyme expression analysis. Total RNA was extracted from liver tissues using TRIzol Reagent (Invitrogen life technologies). Total RNA was used to make cDNA using the Stratascript first strand cDNA synthesis kit (Stratagene). The steady state mRNA level of the ALAS1 was analyzed by quantitative RT-PCR using iQ SYBR green supermix in an iQ5 real-time PCR detection system (Bio-Rad, Hercules, CA). PCR amplification was performed under the following conditions: one cycle of 3 min. at 95°C; followed by 35 cycles of 15 s at 95°C, 30 s at 60°C, 25 s at 72°C, and 10 s at 70°C, 10 s at 75°C and 30 s at 80°C (detection temperature of 82°C); followed by a single final extension cycle of 72°C for 4 min. Relative transcript level was determined using primers annealing specific cDNA sequences of ALAS1 (forward primer: 5′-CAAAGAAACCCCTCCAGCCAA -3′, reverse primer: 5′-GCTGTGTGCCGTCTGGAGTCTGTG -3′, product length:101 bp). The amount of ALAS1 transcript was calculated as the n-fold difference relative to the control gene actin as an internal control (forward primer: 5′-CGCGTCCACCCGCGAG -3′, reverse primer: 5′-CCTGGTGCCTAGGGCG -3′, product length: 193 bp). Results were expressed according to the formula 2^ΔCt(Actin)−ΔCt(gene)^, where ΔCt represents the difference in threshold cycle between the target and control genes.

Histology and histochemical staining. Formalin-fixed paraffin embedded sections (5 µm) of right and left kidneys were processed for hematoxylin and eosin and sirius red stainings. Lesions and prognostic score of renal biopsies were evaluated by a trained pathologist (ES) using a semiquantitative score adapted from the Banff's consensus criteria for the evaluation of renal allograft biopsies (doi: 10.1111/j.1600-6143.2006.01688.x). Briefly, glomerular, tubulointerstitial and vascular lesions were scored (0 = absence; 1 = mild; 2 = moderate; 3 = severe). The main features scored were: chronic glomerulopathy, tubulitis (t), tubular atrophy (a), interstitial inflammation (i), interstitial fibrosis (sirius red positive) (f), vasculitis (v), subendothelial thickening (th), and arteriolar hyalinosis (ah). In addition, presence of crystals or deposits was ascertained, and occasional findings were reported under ‘observations’.

## Supporting Information

Figure S1 **Expression levels of ALAD and PBGD in the liver of AIP mice suffering from different degrees of renal insufficiency.** The cDNA samples were obtained as described in Materials and Methods section. Quantitative real-time PCR were performed using primers annealing specific cDNA sequences of ALAD (forward primer: 5′-ACGTCTGCTTGTGCCCCTAC -3′, reverse primer: 5′-ACAGCGTCGGTCTCCAAAAG -3′, product length: 311 bp) or PBGD (forward primer: 5′-CACTGCCCGTAACATTCCAA -3′, reverse primer: 5′-GCAACATCCAGGATGTTCTTG -3′, product length: 107 bp). The amount of each transcript was calculated as the n-fold difference relative to the control gene actin as an internal control (forward primer: 5′-CGCGTCCACCCGCGAG -3′, reverse primer: 5′-CCTGGTGCCTAGGGCG -3′, product length: 193 bp). The amount of each transcript was expressed according to the formula 2^ΔCt(Actin)−ΔCt(gene)^, where ΔCt represents the difference in threshold cycle between the target and control genes. The non-parametric Mann–Whitney U-test was used for comparison of two groups of mice. Nx5/6, 2/3 nephrectomy of the left kidney and extirpation of the right kidney; Nx 6/6, total nephrectomy. ALAD, aminolevulinate acid dehydratase; PBGD, porphobilinogen deaminase; AIP, Acute Intermittent Porphyria.
(TIF)

Figure S2 **Lack of histological abnormalities in the remnant kidney pole from porphyric mice one month after 2/3 nephrectomy of the left kidney and extirpation of the right kidney.** A) This image indicates well organized histoarchitecture of the renal cortex from a porphyric mice. Glomerulus (arrow) was surrounded by glomerular capsule including proximal (*) and distal tubules (#), B) Cross sections of tubules in the medulla. Kidney samples were taken three days after the last phenobarbital dose. No heme precursor deposits or vascular atrophy were observed (periodic acid-Schiff stain, magnification ×200).
(TIF)

Figure S3 **Unchanged hepatic PBGD protein level in wild type and AIP mice ten hours after total nephrectomy.** Male mice data are presented in left panel and female animals in the right panel. Immunoblot assay was performed as described [13]. Briefly, total liver proteins (50 µg/lane) were resolved by electrophoresis on a 12% polyacrylamide gel and blotted onto PVDF membranes (Amersham HybondTM-P, Buckimghamshire.UK). After blocking, the membranes were incubated with primary antibodies against human PBGD (1∶5000, rabbit polyclonal anti-hPBGD) or GAPDH antibodies (1∶5000, AbD SEROTEC, Oxford. UK). Secondary antibodies used were anti- rabbit (1∶5000, GAR, Biorad) or anti mouse (1∶5000, GAM, Pierce-Rockford. IL), respectively. The signals were then visualized using the Western Lightning Chemiluminescence Reagent Plus (PerkinElmer LAS, Boston). Immunoblot analysis of hepatic PBGD. Densitometry quantifications were performed for two independent immunoblots. The non-parametric Mann–Whitney U-test was used for comparison of two groups of mice. PBGD, porphobilinogen deaminase; AIP, Acute Intermittent Porphyria.
(TIF)
